# Proteomic Analysis Identifies Distinct Protein Patterns for High Ovulation in *FecB* Mutant Small Tail Han Sheep Granulosa Cells

**DOI:** 10.3390/ani14010011

**Published:** 2023-12-19

**Authors:** Xiangyu Wang, Xiaofei Guo, Xiaoyun He, Ran Di, Xiaosheng Zhang, Jinlong Zhang, Mingxing Chu

**Affiliations:** 1 State Key Laboratory of Animal Biotech Breeding, Institute of Animal Science, Chinese Academy of Agricultural Sciences, Beijing 100193, China; wangxiangyu@caas.cn (X.W.); hexiaoyun@caas.cn (X.H.); diran@caas.cn (R.D.); 2 Tianjin Key Laboratory of Animal Molecular Breeding and Biotechnology, Tianjin Engineering Research Center of Animal Healthy Farming, Institute of Animal Science and Veterinary, Tianjin Academy of Agricultural Sciences, Tianjin 300381, China; guoxfnongda@163.com (X.G.); zhangxs0221@126.com (X.Z.); jlzhang1010@163.com (J.Z.); 3 Jilin Provincial Key Laboratory of Grassland Farming, Jilin Province Feed Processing and Ruminant Precision Breeding Cross Regional Cooperation Technology Innovation Center, Northeast Institute of Geography and Agroecology, Chinese Academy of Sciences, Changchun 130102, China

**Keywords:** Small Tail Han sheep, fertility, *FecB* mutation, follicular granulosa cells, proteome

## Abstract

**Simple Summary:**

Our study aimed to investigate the impact of the *FecB* mutation on ovulation in sheep. We analyzed the proteomic profiles of granulosa cells from wild-type, heterozygous, and mutant homozygous sheep. Through our analysis, we identified 199 proteins that showed differential abundance and were involved in important metabolic pathways related to oocyte development. These findings provide insights into the processes of oocyte conservation during follicular development and highlight the role of nutrient metabolism in oocyte maturation. Additionally, we introduced a heterozygous sheep to study the additive effect of the *FecB* mutation on ovulation. By using advanced clustering and machine learning algorithms, we identified potential biomarkers associated with multi-ovulation. These biomarkers, such as ZP2, ZP3, and APOA1 proteins, have the potential to assess oocyte quality and regulate hormone synthesis, thereby controlling ovulation number. Overall, our study contributes to the understanding of how *FecB* mutations affect ovulation in sheep and has implications for sheep breeding.

**Abstract:**

The *Booroola* fecundity (*FecB*) mutation in the bone morphogenetic protein receptor type 1B (BMPR1B) gene increases ovulation in sheep. However, its effect on follicular maturation is not fully understood. Therefore, we collected granulosa cells (GCs) at a critical stage of follicle maturation from nine wild-type (*WW*), nine heterozygous *FecB* mutant (*WB*), and nine homozygous *FecB* mutant (*BB*) Small Tail Han sheep. The GCs of three ewes were selected at random from each genotype and consolidated into a single group, yielding a total of nine groups (three groups per genotype) for proteomic analysis. The tandem mass tag technique was utilized to ascertain the specific proteins linked to multiple ovulation in the various *FecB* genotypes. Using a general linear model, we identified 199 proteins significantly affected by the *FecB* mutation with the LIMMA package (*p* < 0.05). The differential abundance of proteins was enriched in pathways related to cholesterol metabolism, carbohydrate metabolism, amino acid biosynthesis, and glutathione metabolism. These pathways are involved in important processes for GC-regulated ‘conservation’ of oocyte maturation. Further, the sparse partial least-squares discriminant analysis and the Fuzzy-C-mean clustering method were combined to estimate weights and cluster differential abundance proteins according to ovulation to screen important ovulation-related proteins. Among them, ZP2 and ZP3 were found to be enriched in the cellular component catalog term “egg coat”, as well as some apolipoproteins, such as APOA1, APOA2, and APOA4, enriched in several Gene Ontology terms related to cholesterol metabolism and lipoprotein transport. A higher abundance of these essential proteins for oocyte maturation was observed in BB and WB genotypes compared with WW ewes. These proteins had a high weight in the model for discriminating sheep with different *FecB* genotypes. These findings provide new insight that the *FecB* mutant in GCs improves nutrient metabolism, leading to better oocyte maturation by altering the abundance of important proteins (ZP2, ZP3, and APOA1) in favor of increased ovulation or better oocyte quality.

## 1. Introduction

The *Booroola* fecundity (*FecB*) mutation was the first causal mutation to be localized that affects fecundity in sheep. This mutation causes a glutamine to arginine substitution at position 249 of the bone morphogenetic protein receptor type 1 B (BMPR1B) protein (chr6: p.Q249R) [1,2,3]. Recently, sheep constructed with the *FecB* mutation using gene editing techniques demonstrated that the *FecB* mutation significantly increased the litter size of low-fertility sheep [4,5]. Our research previously found that the *FecB* mutation significantly increased ovulation and litter sizes in Small Tail Han (STH) sheep [6]. The *FecB* mutation had a partially dominant effect on lambing number [7] and an additive effect on the number of ovulations [8].

In Booroola sheep, antral follicles had a smaller diameter and an increased oocyte/follicle diameter ratio in homozygote *FecB* mutants (*BB*) versus wild-type ewes (*WW*) [3]. McNatty et al. [9] found that *FecB* mutant ewes had mature follicles with reduced diameter and fewer granulosa cells contained in antral follicles compared with wild-type ewes. Reader et al. [10] found that the primary follicle diameter, area of follicle surface-bound granulosa cells, and number of ribosomes, smooth endoplasmic reticulum, and mitochondria were increased in mutant ewes relative to wild-type sheep. Our results revealed that STH sheep possessing the *FecB* mutation ovulated earlier in estrus onset than the *WW* ewes and had mature follicles with smaller diameters than the *WW* ewes [11]. Therefore, we suggested that the *FecB* mutation has an impact on the proliferation of granulosa cells during the development of follicles without hindering follicular growth. Additionally, this mutation accelerates the differentiation of granulosa cells and promotes oocyte maturation, ultimately leading to a higher occurrence of dominant follicles.

Ovarian granulosa cells, as the main functional cells of ovarian tissue, are wrapped around the oocyte and involved in follicle formation, development, and maintenance of ovarian function through the secretion of steroid hormones [12]. Granulosa cells undergo proliferation and progressive differentiation throughout ovarian follicular development to facilitate the maturation of oocytes and subsequent ovulation [13]. It has been demonstrated that follicular atresia is due to the apoptosis of granulosa cells, resulting in a decrease in the number of follicles [14,15]. The BMP pathway to which BMPR1B belongs plays an important role in oocyte–granulosa cell interactions [16,17]. It has been shown that partial inactivation of the *FecB* gene in Australian *Booroola* ewes carrying the *BB* genotype resulted in earlier differentiation of granulosa cells and earlier maturation of ovulated follicles compared with non-carriers [1]. Although there are transcriptomic and proteomic studies on ovaries with different genotypes of *FecB* [18,19], the important role of granulosa cell factors in follicle development and ovulation with the *FecB* mutation cannot be clarified by large-scale omics studies of ovaries alone, largely because of the coexistence of ovarian follicles at different developmental stages and the obvious functional differences between oocytes and granulosa cells in one follicle. Studies on the follicular granulosa cell proteome in pigs, mice, and humans have expanded our knowledge of the role of granulosa cells in follicular development and maturation [20,21,22,23].

The *FecB* mutation results in a partial functional deficiency of the BMPR1B protein, which has been found to impact folliculogenesis and ovulation rates in sheep. However, our knowledge regarding the influence of this mutation on oocyte survival and morphology remains limited [24]. Granulosa cells play an essential role in follicular development and ovulation. Therefore, this study aimed to investigate the proteome of granulosa cells at a crucial stage of follicle maturation in sheep with different *FecB* genotypes. The proteome profiles were constructed using tandem mass tagging (TMT) and parallel reaction monitoring (PRM) methods. Furthermore, a general linear model combined with a machine learning strategy were used to analyze proteomic data to screen core proteins and key signaling pathways in granulosa cells that regulate ovulation in the presence of the *FecB* mutation.

## 2. Materials and Methods

### 2.1. Experimental Design and Sample Preparation

**Figure 1:**
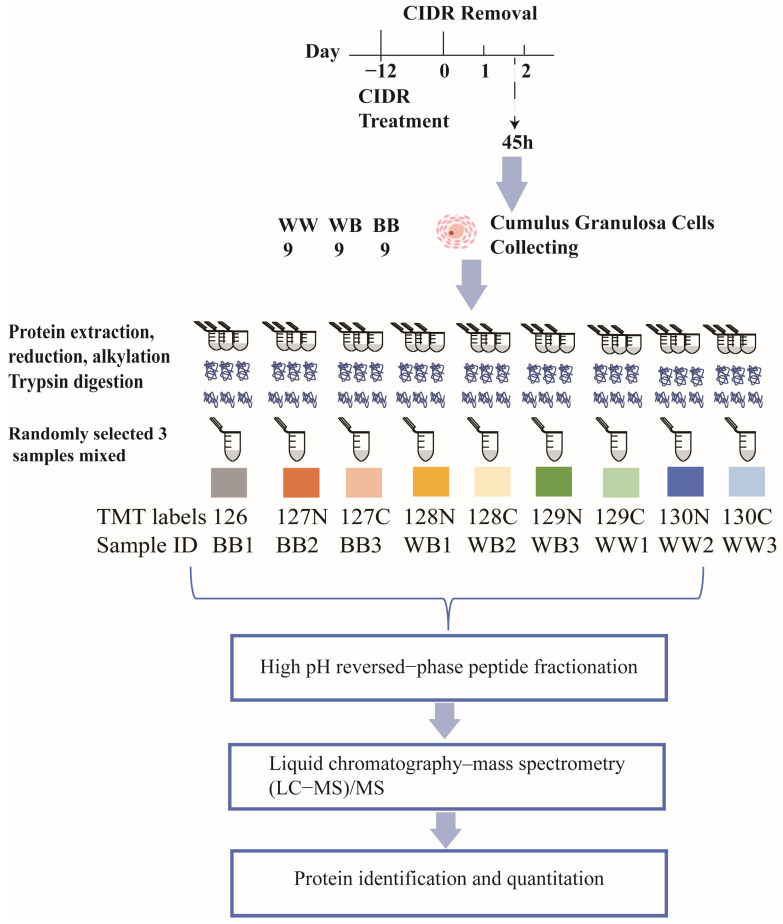
Description of the process of collecting granular cells and conducting TMT proteome analysis. A total of 27 ewes, divided into three groups based on their *FecB* genotype (9 *WW*, 9 *WB*, and 9 *BB*), were subjected to controlled internal drug release (CIDR) to synchronize estrus. Cumulus-oocyte complexes (COCs) were obtained from follicles larger than 3 mm in diameter, 45 h after CIDR removal on day 12. Following the extraction of cumulus granulosa cells, proteins were isolated and randomly distributed into three groups for TMT labeling.

A cohort of 27 ewes, approximately 3 years old and with comparable body weights, were chosen from the STH nucleus flock located in Shandong Province, China. The group of ewes comprised 9 *WW* ewes, 9 *WB* ewes, and 9 *BB* ewes. Notably, there were significant differences in litter size and ovulation among different *FecB* genotypes (Table 1). The ewes were raised in natural light and fed ad libitum. Based on our previous study protocols, a controlled internal drug release device (Pfizer Animal Health, Auckland, New Zealand) was employed to induce simultaneous estrus in a group of 27 ewes. Subsequently, antral follicle diameters were observed laparoscopically at 45 h following the removal of the controlled internal drug release device on day 12. Using a syringe filled with Dulbecco’s phosphate-buffered saline (Thermo Fisher Scientific, Waltham, MA, USA), cumulus-oocyte complexes (COCs) were obtained through aspiration from follicles that had a diameter exceeding 3 mm [6,11]. Then, a total of 27 COCs derived from three different genotypes (9 *WW*, 9 *WB*, and 9 *BB*) underwent washing using Dulbecco’s phosphate-buffered saline and subsequent digestion with hyaluronidase. Following a 5 min centrifugation at 400× *g*, the cumulus granulosa cells were immediately frozen in liquid nitrogen and preserved at a temperature of −80 °C for subsequent protein extraction (Figure 1).

### 2.2. Peptide Preparation and TMT Labeling

The protein samples from cumulus cells collected from the 27 COCs were separately extracted using the SDT lysis technique [25]. To determine protein concentrations, the Pierce™ BCA Protein Assay Kit (Thermo Fisher Scientific, USA) based on bicinchoninic acid was employed [26]. Each sample had around 100 µg of protein extracted and then digested using trypsin, employing a sample preparation method that involved a filter-aided technique [27]. For further proteomic experiments (Figure 1), the amount of peptides was measured using ultraviolet spectrophotometry at an optical density of 280. To comply with the sample size specifications of the TMT labeling kit, out of the nine samples obtained from each genotype, three samples (each containing 50 μg) were randomly chosen and combined to form a single sample for peptide labeling. Consequently, a total of nine mixed samples (comprising three samples for each genotype) were acquired for the three genotypes. These nine samples (100 μg per sample) were then labeled with peptides using a 10-fold tandem mass labeling with the TMT mass labeling kit (Thermo Fisher Scientific, USA). Specifically, the *WW* genotype samples were labeled with 129C, 130N, and 130C, the *BB* genotype samples were labeled with 126, 127N, and 127C, and the markers for the *WB* genotype samples were 128N, 128C, and 129N, respectively (Figure 1).

### 2.3. Analysis of Peptide Fractionation and Liquid Chromatography–Mass Spectrometry/Mass Spectrometry (LC-MS/MS) Using Various Methods

Nine samples containing labeled peptides were combined in equal amounts. Then, 100 μg of lyophilized mixed peptides were diluted with 0.1% trifluoroacetic acid to a volume of 300 µL. The mixture was then fractionated into 10 fractions using the Pierce high-pH reversed-phase fractionation kit (Thermo Fisher Scientific, USA). Afterwards, a total of 10 peptide fractions were separated using a step-gradient method with increasing concentrations of acetonitrile. Following the process of vacuum drying, the eluted samples were subsequently re-solubilized using a solution containing 0.1% formic acid. The concentration was then assessed by measuring the absorbance at an optical density of 280.

The proportions of each specimen were loaded onto the Easy nLC nanoflow liquid chromatography device (Thermo Fisher Scientific, USA) for high-performance liquid chromatography separation. At first, the samples were placed onto a reverse phase trap column called C18 Acclaim PepMap100, which had dimensions of 100 μm × 2 cm and was manufactured by Thermo Fisher Scientific in the United States. Subsequently, the samples were separated using an analytical column known as EASY-Column C18-A2, which was a reversed-phase analytical column measuring 10 cm in length and 75 μm in inner diameter. This column contained 3 μm resin and was also produced by Thermo Fisher Scientific in the USA. The buffer system consisted of buffer A, which contained 0.1% formic acid, and buffer B, consisting of 0.1% formic acid in 84% acetonitrile. To prepare the chromatographic column, a solution containing 95% buffer A was used for equilibration. The implementation of the linear gradient strategy involved a 1 h gradient where buffer B was gradually increased from 0% to 50% over a period of 50 min. This was then followed by a 5 min transition to a gradient of 50–100% buffer B. Finally, the system maintained a steady state with 100% buffer B for an additional 5 min. IntelliFlow technology regulates the flow rate at 300 nanoliters per minute.

Positive ion mode was used to operate a Thermo Scientific Q Exactive Mass Spectrometer (Thermo Fisher Scientific, USA) for the analysis of Mass spectrometry (MS). The acquisition of the MS data involved employing a data-dependent approach that utilized a top-20 method. This method dynamically chose the most prevalent precursor ions from the survey scan (300–1800 *m*/*z*) to undergo higher energy dissociation (HCD) fragmentation. The survey scan has a mass resolution of 70,000 at 200 *m*/*z*, with an automatic gain control (AGC) target of 1 × 10^6^. The maximum injection time is set at 50 ms, and a dynamic exclusion time of 60 s is implemented. For the HCD spectra resolution, it is set at 35,000 at 200 *m*/*z*. An isolation window of 2 *m*/*z* is applied, along with a normalized collision energy of 30 eV. Additionally, an underfill ratio of 0.1% has been established. Peptide recognition mode was used to operate the instrument.

### 2.4. Protein Identification and Quantification Analysis

Proteome Discoverer v.1.4 (Thermo Fisher Scientific, USA) and MASCOT engine v.2.2 (Matrix Science, London, UK) were utilized to analyze the MS spectra data. Proteins were identified using the uniprot_Ovis_aries_28089_20190905.fasta protein database. This sheep protein database was downloaded from the Uniprot database and contains 28,089 protein sequences. Supplementary Table S1 displays the search parameter configurations.

To determine the differences in protein abundance among the groups, proteins were filtered if their abundance was not quantified for at least two samples within any group. The log2-transformed and normalized abundance of filtered proteins were calculated using the linear models for microarray data (LIMMA v 3.52.1), employing a factorial design with genotype as a factor (*WW*, *WB*, and *BB*) [28]. For screening differentially abundant proteins (DAPs) between the two groups, the criteria used were a fold change greater than 1.2 or less than 0.83 and a *p* value lower than 0.05, as explained in a previous study [29].

To investigate the trend of key proteins affected by the *FecB* mutation in granulosa cells as the allele number of the *FecB* mutation increases, we clustered the DAPs using the Fuzzy c-means clustering algorithm. Sparse partial least-squares discriminant analysis (sPLSDA) of the mixOmics (v.6.20.0) R package was used to analyze the weights of DAPs in different genotypes to select the key proteins affected by the *FecB* mutation [30]. For tuning the model, the optimum number of components was determined using Leave-One-Out validation with the perf() function and the tune. The splsda() function was used to estimate the number of variables in each potential component. The model’s performance was evaluated with area under the curve scores.

### 2.5. Functional Annotation and Enrichment Analysis of Key Proteins

In order to investigate the possible involvement of these proteins in the influence of FecB mutations on fertility, clusterProfiler (v4.4.4) [31] on Gene Ontology (GO) categories [32] and Kyoto Encyclopedia of Genes and Genomes (KEGG) [33]. The pAdjustMethod was used for false discovery rate analysis. Top-enriched GO categories and KEGG pathways were visualized using the web tool ChiPlot (https://www.chiplot.online/, accessed on 27 March 2023). Interaction relationships between DAPs were searched using the STRING (http://string-db.org/) database with default parameters, except confidence at ≥0.4. To explore the potential role of these proteins in the impact of *FecB* mutations upon fertility, over-representation analyses were run by clusterProfiler (v4.4.4). The pAdjustMethod was used for false discovery rate analysis. Top-enriched KEGG pathways and GO categories were visualized using the web tool ChiPlot (https://www.chiplot.online/, accessed on 24 April 2023). Interaction relationships between DAPs were searched using the *STRING* (http://string-db.org/, accessed on 24 April 2023) database with default parameters, except confidence at ≥0.4.

### 2.6. Quantitative Analysis of Selected Proteins with PRM

In order to verify the protein levels obtained through TMT quantification, PRM analysis was utilized to quantify the protein abundances in granulosa cells [34]. Tryptic fragments were generated using the procedure outlined in the TMT investigation. Next, 1 femtomole of peptides with stable isotopes (IGDYAGIK) from Thermo Fisher Scientific in the United States was included as a calibration peptide. These calibration peptides were added to 1 microgram of sample peptides to serve as an internal standard reference. The peptides utilized for PRM can be found in Table S2. Prior to conducting reversed-phase chromatography on an Easy nLC-1200 device manufactured by Thermo Fisher Scientific in the United States, peptide mixtures underwent desalting using self-made C18 stage tips with an inner diameter of 75 μm and a resin of 3 μm. The solutions A and B in the liquid phase were identical to those mentioned in the aforementioned TMT procedure. The 1 h gradient of liquid chromatography was as below: the concentration of solution B ranged from 5% to 10% for 0–2 min, from 10% to 30% for 2–45 min, from 30% to 100% for 45–55 min, and then was maintained at 100% for 55–60 min. A Q-active HF mass spectrometer (Thermo Fisher Scientific, USA) was utilized to conduct the PRM analysis. The mass spectrometer was used in the positive ion mode for one hour, employing the following settings: a full MS1 scan was obtained with a resolution of 60,000 (at 200 *m*/*z*), AGC target values were set at 3 × 10^6^, and the maximum injection time was 200 ms. After conducting full MS scans, 20 PRM scans were performed at a resolution of 30,000 (at 200 *m*/*z*), maintaining AGC at 3 × 10^6^ and a maximum injection time of 120 ms. Peptides of interest were extracted using a window size of 1.6 Th. Ion activation/dissociation were performed at a normalized collision energy of 27 in a HCD collision cell. Skyline v.3.5.0 was utilized to extract the peptide peak areas for each peptide. Subsequently, the relative peptide peak areas of each sample were integrated and then normalized with the peak areas of calibration peptides. Skyline v.3.5.0 was utilized to extract the peptide peak areas for each peptide. Subsequently, the relative peptide peak areas of each sample were integrated and then normalized with the peak areas of calibration peptides [35].

## 3. Results

### 3.1. Protein Identification and Profiling

The experiments utilizing TMT-based techniques successfully validated 23,454 spectra that matched peptides. Additionally, 9406 peptides were identified, out of which 8043 were unique. Ultimately, a total of 2314 proteins were identified in all samples, each represented by at least one unique peptide. Furthermore, over 60% of the identified proteins contained multiple unique peptides (Supplementary Table S2). The results of the peptide ionscore indicated that over 70.5% of the peptides achieved a score exceeding 20, with a median score of approximately 28.61 (Supplementary Figure S1A). This suggests that the acquired data possesses a relatively high level of quality. The majority of the identified peptides exhibited lengths ranging from 5 to 17 amino acids (Supplementary Figure S1C). The analysis of protein molecular weight distribution showed that the molecular weights of 99% of proteins were between 6 and 312 kDa (Supplementary Figure S1B).

### 3.2. Data Validation

**Figure 2:**
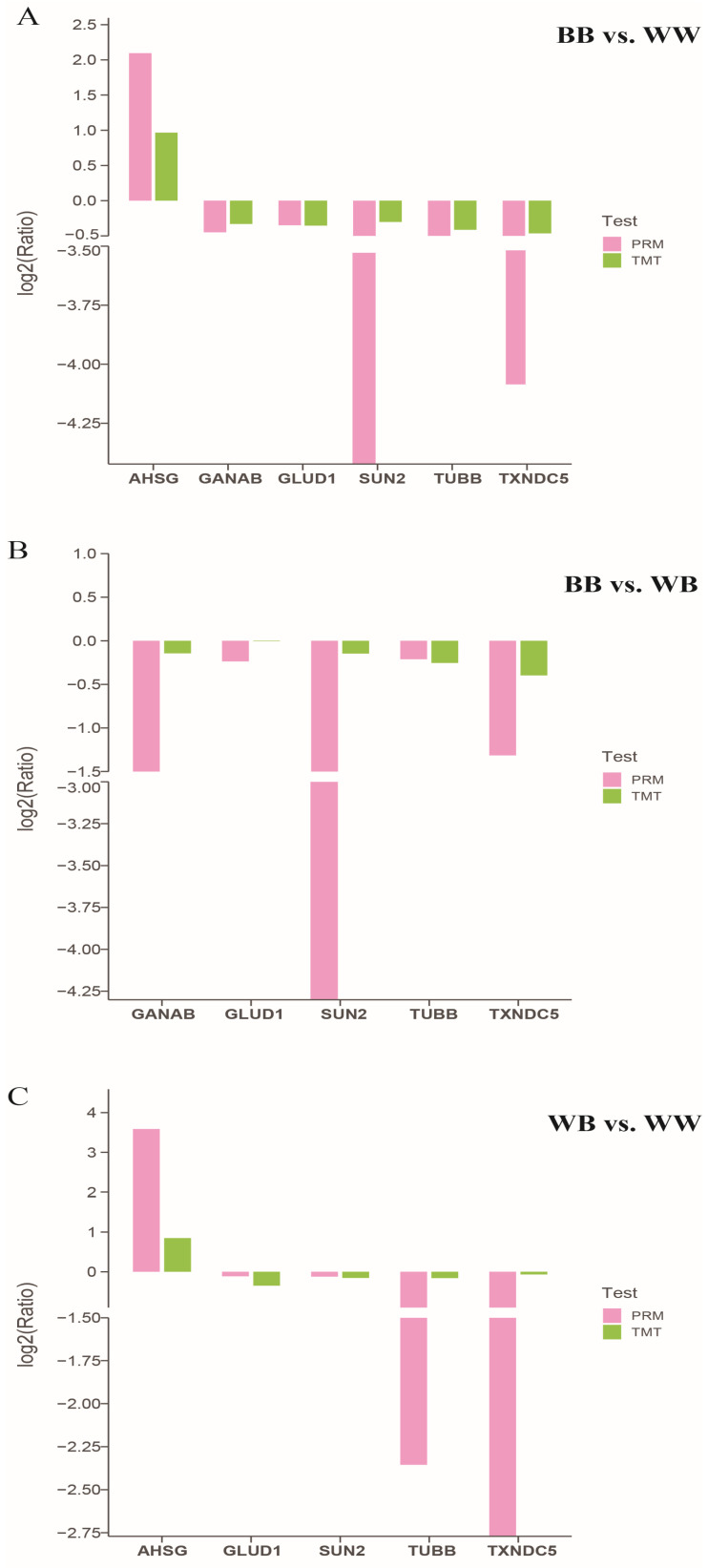
Illustration of the comparison of protein abundance profiles obtained through the employment of PRM and TMT protein quantification techniques. In the comparison of *BB* ewes with *WW* ewes (*BB* vs. *WW*), *BB* ewes with *WB* ewes (*BB* vs. *WB*), and *WB* ewes with *WW* ewes (*WB* vs. *WW*) groups, the following proteins were chosen for validation of their abundance: alpha 2-HS-glycoprotein (AHSG), glutamate dehydrogenase (GLUD1), tubulin beta chain (TUBB), glucosidase II alpha subunit (GANAB), SUN domain-containing protein (SUN2), and thioredoxin domain containing 5 (TXNDC5). The log2(Ratio) values were computed for the compared genotypes. The protein abundance, as determined by the PRM method in comparison to the TMT method, exhibited a consistent pattern for the selected genes across all three groups.

Based on protein abundance across various groups in the TMT analysis, a subset of differentially abundant proteins was chosen to corroborate the TMT findings through the employment of the PRM technique. In all comparisons between the *BB* vs. *WB*, *BB* vs. *WW*, and *WB* vs. *WW* groups, the following genes were chosen for PRM analysis: alpha 2-HS-glycoprotein (AHSG), glucosidase II alpha subunit (GANAB), glutamate dehydrogenase (GLUD1), SUN domain-containing protein (SUN2), tubulin beta chain (TUBB), and thioredoxin domain containing 5 (TXNDC5). Our findings indicated that the PRM results for these selected genes exhibited similar directional abundance trends (both up and down) as the TMT results observed in the various groups (Figure 2 and Supplementary Table S3).

### 3.3. Global Differential Protein Abundance and Annotation

**Figure 3:**
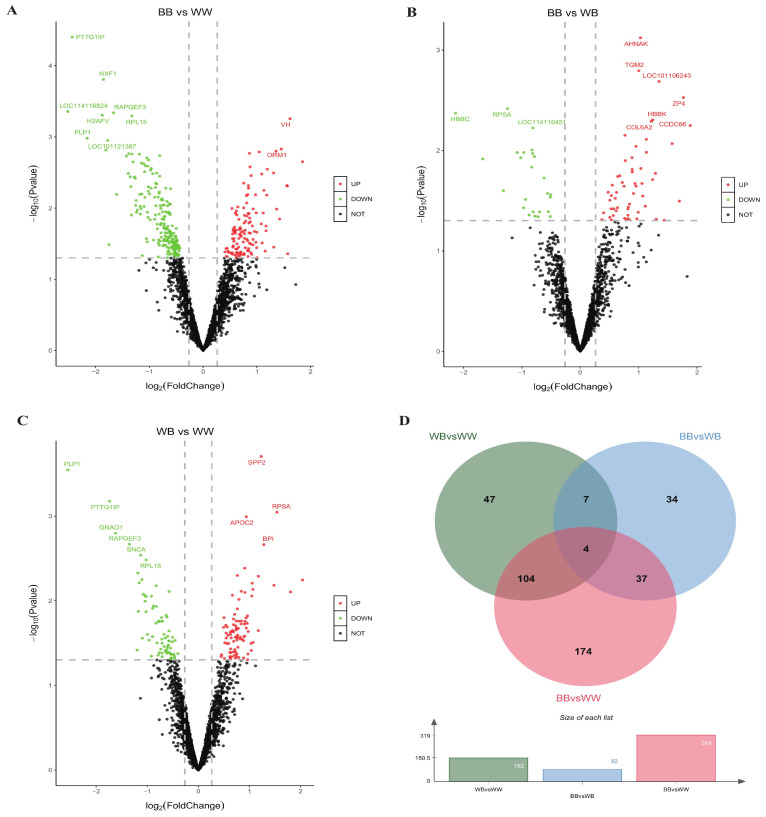
The volcano plots and Venn plots depict the differentially abundant proteins (DAPs). The threshold for identifying upregulated and downregulated DAPs was set at a fold change > 1.2 or <0.83, with a *p* value < 0.05. The separate analysis of DAPs was performed for the (**A**) comparison between *BB* ewes and *WW* ewes (*WW* vs. *BB*), (**B**) comparison between *BB* ewes and *WB* ewes (*BB* vs. *WB*), and (**C**) comparison between *WB* ewes and *WW* ewes (*WB* vs. *WW*) groups. Additionally, the Venn plot in (**D**) illustrates the overlap of DAPs between the *BB* vs. *WW*, *BB* vs. *WB*, and *WB* vs. *WW* groups.

A total of 199 DAPs (*p* < 0.05) influenced by the *FecB* mutation were subjected to analysis using LIMMA with the limFit and eBayes functions (Supplementary Table S4). Additionally, contrast tests were conducted to compare different genotypes. Based on the filter criteria of a foldchange > 1.2 or <0.83 and a *p* value < 0.05, 319 DAPs were detected in the *BB* vs. *WW* group, and 123 were upregulated and 196 were downregulated in the *BB* genotype (Figure 3A and Supplementary Table S5). Likewise, 82 DAPs were detected in the *BB* vs. *WB* group, with 25 upregulated and 57 downregulated (Figure 3B and Supplementary Table S6). In the *WB* vs. *WW* group, 162 DAPs were detected, with 91 proteins upregulated and 71 proteins downregulated (Figure 3C and Supplementary Table S7). The overlaps between the three groups were visualized using a web tool (http://jvenn.toulouse.inra.fr/app/example.html, accessed on 21 March 2023) [36]. Four proteins (Q70TH4, W5NTE3, W5PY18, and W5Q7E7) were identified in all three groups (Figure 3D).

**Figure 4:**
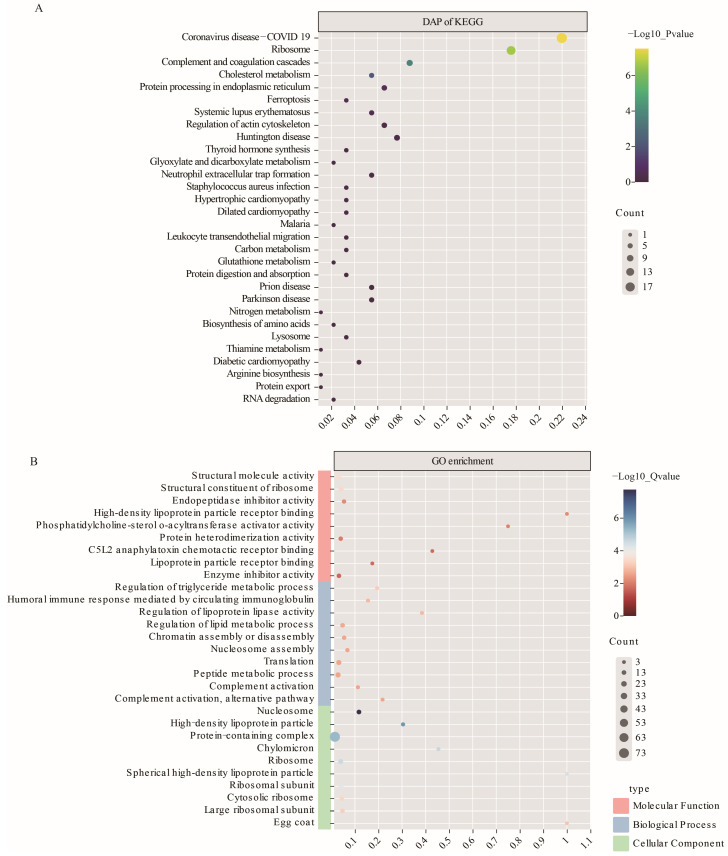
The top 30 enriched KEGG pathways and top 30 Gene Ontology (GO) terms in each category of differentially abundant proteins (DAPs) were obtained through the genotype-based factorial model. (**A**) KEGG enrichment pathways for DAPs. (**B**) GO terms in each category for DAPs.

The DAPs were annotated using KEGG enrichment and GO analysis, as indicated in Supplementary Tables S8 and S9. Figure 4A displays the top 30 KEGG pathways. Notably, pathways such as cholesterol metabolism, thyroid hormone synthesis, carbon metabolism, glutathione metabolism, nitrogen metabolism, and biosynthesis of amino acids were of particular interest because of the significant involvement of associated factors in follicular development, oocyte maturation, and luteinization of growing follicles [11,22,37].

### 3.4. Weighted Analysis of DAPs to Identify Proteins Associated with Prolific STH Sheep

**Figure 5:**
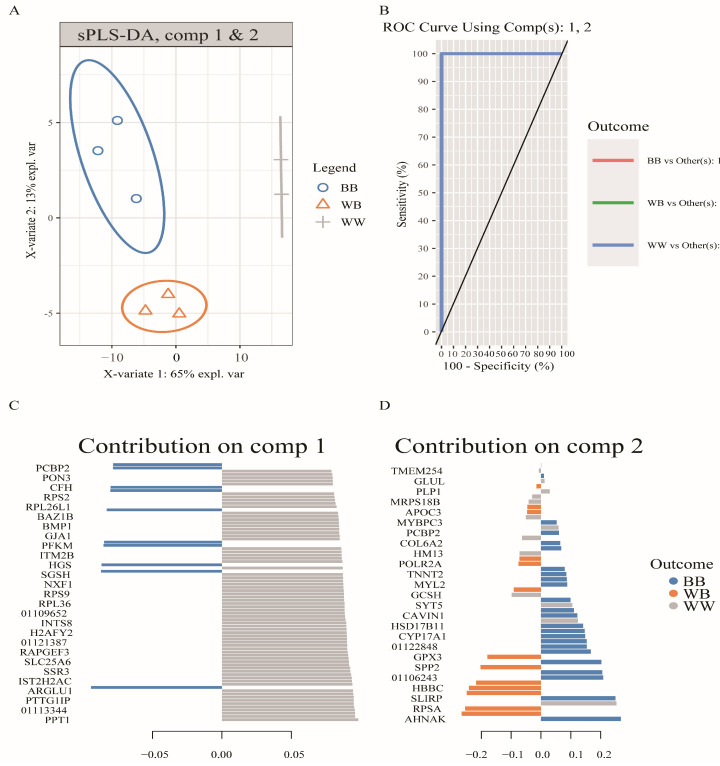
Demonstration of the application of sparse partial least-squares discriminant analysis (sPLSDA) combined with the examination of differentially abundant proteins (DAPs) acquired using a genotype-based factorial model. By employing the sPLSDA method, the impact of mutations on protein abundance was predicted, and the weights of these proteins in various genotypes were calculated. Consequently, a screening process was conducted to identify biomarkers affected by FecB mutations, resulting in the identification of two components. (**A**) The screened proteins exhibited a notable ability to differentiate samples with distinct genotypes. (**B**) The Receiver Operating Characteristic Area under the Curve (ROC AUC) yields separate results of 1.00 when utilizing proteins from the two components within the predictive model. (**C**,**D**) The visualization showcases proteins with higher weights in the two components of the model. The bars represent the absolute values of the calculated loading vector weights. The varying colors indicate the level of association between each biomarker and the genotype. *BB* refers to sheep with the *BB* genotype, *WB* refers to sheep with the *WB* genotype, and *WW* refers to sheep with the *WW* genotype.

To identify the *FecB* mutation-related feature between the three genotypes, the sPLSDA approach was used for the selection of variables in multiclass problems. The results of estimating the classification overall error rate with “centroids.dist” and “max.dist” metrics showed that the sPLSDA model achieved the best classification performance when ncomp = 2 (Figure S2). The scatter plots showed that the protein abundance patterns of type *BB*, *WB*, and *WW* sheep varied considerably between the three genotypes (Figure 5A). The receiver operating characteristic curve plot showed that the model including both components had perfect classification accuracy (Figure 5B). The stability of selected features for each component was calculated and plotted with the perf() function. The first and second components were selected with 180 and 50 discriminant variables, respectively (Supplementary Table S10), and the model prediction error rate was significantly reduced with the addition of the second component variables (Figure S2). The feature variables in each component and their weights are shown in Figure 5C. The combination of characteristic variables in component 1 allowed for discrimination between the wild-type and heterozygotes of the *FecB* mutation, and with the combination of variables in component 2, the *BB* and *WB* genotypes of sheep were distinguished.

**Figure 6:**
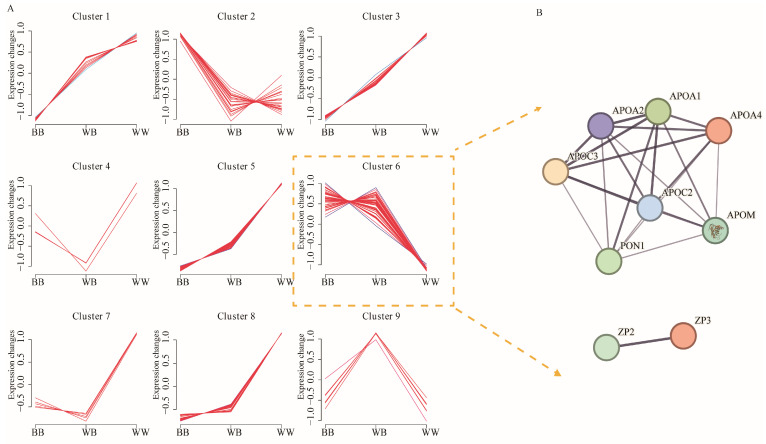
The clustering diagram of differentially abundant proteins (DAPs) and the protein-protein interaction network of the proteins in cluster 6. (**A**) The clustering diagram of DAPs was obtained using the genotype-based factorial model and the fuzzy c-means clustering algorithm. (**B**) The interaction network of the proteins in cluster 6 was constructed using the STRING database. The edges in the network represent both functional and physical protein associations, with the thickness of the confidence lines indicating the strength of data support.

The DAPs affected by the *FecB* mutation were clustered using Fuzzy-C-means clustering (Figure 6 and Supplementary Table S11). A total of 199 DAPs were classified into nine distinct patterns according to their genotypes. Notably, protein abundance in clusters 1 and 3 exhibited a decline as the allele number of the *FecB* mutation increased. Additionally, the protein levels in clusters 5, 7, and 8 were found to be lower in ewes with the *FecB* mutation compared with those with the wild-type genotype. However, no statistically significant difference was observed between ewes homozygous or heterozygous for the *FecB* mutation. Within clusters 5, 7, and 8, the HIST3H2BB, HIST1H1D, H2AFY2, HP1BP3, and BAZ1B proteins exhibited substantial weights in component 1 and were found to be significantly enriched in two distinct GO terms, namely ‘Structural constituent of ribosome’ and ‘Chromatin assembly or disassembly’ (Figure 6). In cluster 6, protein expression was significantly higher in both *BB* and *WB* types than in wild-type ewes. The proteins ZP2 and ZP3, found in cluster 6, exhibited enrichment in the ‘egg coat’ term. Additionally, APOA1, APOA2, APOA4, APOC2, APOC3, APOH, and APOM demonstrated enrichment in various GO terms associated with cholesterol metabolism and lipoprotein transport. The potential interactions of functionally significant proteins within cluster 6 were predicted using the STRING database, as depicted in Figure 6B.

## 4. Discussion

The present study investigated the impact of the *FecB* mutation on ovulation, specifically focusing on its additive effect. To achieve this, we incorporated *FecB* mutation heterozygotes and employed a general linear model in conjunction with a machine learning approach to identify proteins that contribute to increased ovulation in sheep granulosa cells. The multiclass data obtained from this analysis complements previous findings on the influence of an increased allele number of the *FecB* mutation on the proteome of granulosa cells. Consequently, our study offers further understanding of the relationship between the *FecB* mutation and ovulation.

Bone morphogenetic protein 1 (BMP1) is a significant member of the astacin family and is classified as a metalloproteinase [38]. In the current study, sPLSDA analysis revealed that BMP1 had a substantial weighting feature for the classification of distinct *FecB* genotypes. Within sheep antral follicles, granulosa cells are the primary site of BMP1 synthesis [39]. The present proteomic findings for granulosa cells of sheep with various *FecB* genotypes also revealed the presence of BMP1 expression. Previous research on BMP1 demonstrated its involvement in the regulation and modulation of BMP signaling, both directly through prodomain binding and indirectly through chordinase cleavage [40,41]. BMP1 is positioned centrally within a hypothetical feedback loop that orchestrates ligand signaling for the transforming growth factor B (TGFB) superfamily within the ovary. BMP1 regulates signaling via the SMAD2/3 and SMAD1/5/8 pathways, which are employed by TGFB/activin and BMP4, respectively [42]. BMP/TGF factors exert a potent inhibitory influence on progesterone secretion by granulosa cells of antral follicles in ewes. The *FecB* mutation was found to decrease the activity of BMP signaling, resulting in a diminished inhibitory effect of TGF-1, activin A, and BMP4 on progesterone secretion from antral follicles [43]. This suggests that BMP1 plays a crucial role in regulating the production of multiple dominant follicles in the presence of the *FecB* mutation. However, further research is required to explore the association between BMP1 and the BMPR1B-dependent signaling pathway.

In this study, a genotype-based factorial model in LIMMA was used to identify zona pellucida sperm-binding protein 2 (ZP2), ZP3, and ZP4 as DAPs affected by the *FecB* mutation. The ZP proteins are important glycosylated proteins that constitute the zona pellucida (ZP) of mammalian oocytes and are involved in oogenesis, sperm-egg interactions during fertilization, and the prevention of polyspermy. Changes in the structure or function of these proteins can lead to infertility [44]. Human ZP is composed of four glycoproteins: ZP1, ZP2, ZP3, and ZP4 [45]. No ZP1 protein was identified in our current sheep granulosa cell proteome results. In most mammals, both oocytes and granulosa cells are involved in ZP synthesis during oogenesis [17,46], but the expression of ZP proteins is species-specific [47,48]. In mice, the ZP proteins consist of ZP1, ZP2, and ZP3. In pigs and cattle, ZP is composed of ZP2, ZP3, and ZP4, similar to sheep [44,49]. ZP2 and ZP3 are absolutely necessary for the formation of ZP around growing oocytes, and mice homozygous for either *ZP2* or *ZP3* are infertile because of the absence of ZP around the oocyte, ultimately resulting in disruption of egg growth and few mature eggs [50]. In wild European Mouflon sheep, ZP2 and ZP3 were found to be associated with fertility [51]. In Hu sheep, the mutation rs401271989 in ZP3 was associated with high fertility rates [52]. It has been shown that ZP3 is an important biomarker for evaluating oocyte quality [37]. The results of this study showed that ZP2 and ZP3 expression patterns were similar in the *BB* and *WB* genotypes, both significantly higher than in the *WW* genotype, and these two proteins had a high weight in component 1. This finding implies that the impacts of ZP3 and ZP4 on the processes of egg maturation and ovulation could potentially be attributed to nonadditive genetic effects. Therefore, we suggest that these two proteins have important roles in the effect of the *FecB* mutation upon ovulation.

Among the DAPs identified in the present study, APOA1, APOA2, APOA4, APOC2, APOC3, APOH, and APOM were enriched in the cholesterol metabolism pathway via KEGG pathway enrichment analysis. Apolipoproteins A1 and A2 are major components of high-density lipoprotein (HDL), and APOC2 and APOC3 are also involved in HDL composition. APOA1 and APOA4 activate lecithin-cholesterol acyltransferase involved in HDL-mediated reverse cholesterol transport. APOH and APOM are also associated with HDL function [53]. It has been reported that abnormal metabolism of HDL and its lipoprotein mutation affect fertility in females and also affect the maturation and embryo quality of oocytes matured in vitro [54,55,56,57]. HDL is the only major lipoprotein detected in follicular fluid, and HDL plays an important role in ovarian cholesterol transport [58]. The process of progesterone and estrogen synthesis in ovarian granulosa cells is dependent on HDL for the uptake of cholesterol as an essential synthetic raw material [59,60]. Oocytes need to accumulate large amounts of mRNA and protein during growth to prepare for post-fertilization embryonic development. To reduce their own metabolic stress, oocytes outsource tasks to their surrounding granulosa cells. Because of the low expression of cholesterol synthesis-related enzymes in oocytes and the lack of HDL and LDL cholesterol receptors, oocytes lack intracellular cholesterol synthesis and extracellular cholesterol acquisition and are dependent on granulosa cells to synthesize and transfer cholesterol to them in the paracrine interaction [61]. Metabolic cooperation between granulosa cells and oocytes ensures that cholesterol is deposited into the oocyte, providing a critical pathway allowing the oocyte to develop into embryos [62,63]. The apolipoproteins detected in the current proteome data were clustered in cluster 6, and the abundance of these proteins was significantly higher in both *BB* and *WB* types than in wild-type ewes. These results are consistent with those reported for the ovarian proteome of ewes with different genotypes of *FecB* [19], suggesting that *FecB* mutant ewes can provide sufficient cholesterol for follicle development and thus produce more ovulated oocytes. It has been shown that increased paraoxonase 1 (PON1) and APOA1 can enhance the resistance of bovine oocytes to oxidative stress and improve embryo survival after in vitro fertilization and maturation of oocytes [57,64]. In the present study, it was found that PON1 and APOA1 were clustered in cluster 6, and the abundances of these proteins were significantly higher in both *BB* and *WB* types than in wild-type ewes. Our previous results on the metabolome of follicular fluid in ewes with different *FecB* genotypes at 45 h after simultaneous estrus withdrawal showed that ewes with the *FecB* mutation had a higher capacity for glutathione metabolism than the wild-type genotype [11]. So, the present results indicated that ewes with the *FecB* mutation have a stronger resistance to oxidative stress by removing the large amount of reactive oxygen species produced during follicular development and maturation.

## 5. Conclusions

The TMT proteome analysis was employed to determine the abundance of protein profiles in granulosa cells during the follicular phase of sheep with the *FecB* mutation. By utilizing analysis of variance and a machine learning classification model, this study successfully identified distinct protein patterns that were associated with increased ovulation in sheep with the *FecB* mutation. The ZP2 and ZP3 proteins potentially hold significance in the process of oocyte maturation in the presence of the *FecB* mutation. Additionally, APOA1 likely participates in the regulation of gonadal hormone synthesis via cholesterol metabolism, enhances the resistance of granulosa cells to oxidative stress, and governs the number of ovulations. The findings of our study demonstrate that the *FecB* mutation is a crucial molecular biomarker for enhancing sheep fertility and breeding. Furthermore, our proteomic analysis of granulosa cells offers novel perspectives on the mechanisms underlying the impact of *FecB* mutations on fertility via granulosa cells.

## Figures and Tables

**Table 1:** Reproduction trait and sample information of the ewes with different *FecB* genotypes.

Genotype	Litter Size	Ovulation Number	Age (Years)	Body Weight (kg)
*BB*	2.44 ± 0.18 ^a1^	2.89 ± 0.39 ^a^	2.78 ± 0.15	73.78 ± 2.63
*WB*	2.33 ± 0.24 ^a^	2.11 ± 0.11 ^b^	2.28 ± 0.18	74.11 ± 3.37
*WW*	1.11 ± 0.11 ^b^	1.11 ± 0.11 ^c^	2.22 ± 0.18	73.667 ± 3.38

^1^ The data were represented as mean ± standard error. Different letters indicate significant differences between different FecB genotypes (*p* < 0.05).

## Data Availability

The data reported in this work have been deposited in the OMIX, China National Center for Bioinformation/Beijing Institute of Genomics, Chinese Academy of Sciences [65,66] (https://ngdc.cncb.ac.cn/omix: accession no.OMIX004762, accessed on 16 August 2023).

## Supplementary Materials

The following supporting information can be downloaded at: https://www.mdpi.com/article/10.3390/ani14010011/s1, Figure S1: Distributions of peptide ion score, molecular weight, and peptide length in the granulosa cells. (A) Distribution of peptide ion score. (B) Protein molecular weight (kDa) distribution. (C) Distribution of peptide length; Figure S2: depicts the visualization of sparse partial least-squares discriminant analysis (sPLSDA). (A) Classification performance per component (overall and balanced error rate) for prediction distances (‘centroids dist’ and ‘max.dist’) using Leave-One-Out validation. (B) The number of variables in each potential component is estimated by the tune.splsda() function; Table S1: The parameters for searching MS data; Table S2: A list of the identified proteins in granular cell; Table S3: The quantification results of PRM assay of selected proteins in different groups; Table S4: The factorial model identified proteins that were significantly changed in abundance by the *FecB* mutation; Table S5: The results of differential proteins abundance in *WB* vs. *WW* group; Table S6: The results of differential proteins abundance in *BB* vs. *WB* group; Table S7: The results of differential proteins abundance in *BB* vs. *WW* group; Table S8: KEGG enrichment analysis in the proteins selected by the factorial model; Table S9: Gene ontology (GO) enrichment analysis for the proteins selected by the factorial model; Table S10: The weight of proteins selected by sPLSDA method in the proteins selected by the factorial model; Table S11: Proteins clustered by Fuzzy-C-means clustering method.
