# Supplementary figures and images for: Proteomic Analysis Identifies Distinct Protein Patterns for High Ovulation in FecB Mutant Small Tail Han Sheep Granulosa Cells
Source: Animals (Basel). 2023 Dec 19;14(1):11. doi: 10.3390/ani14010011 (PMC10778137; doi:10.3390/ani14010011)

A

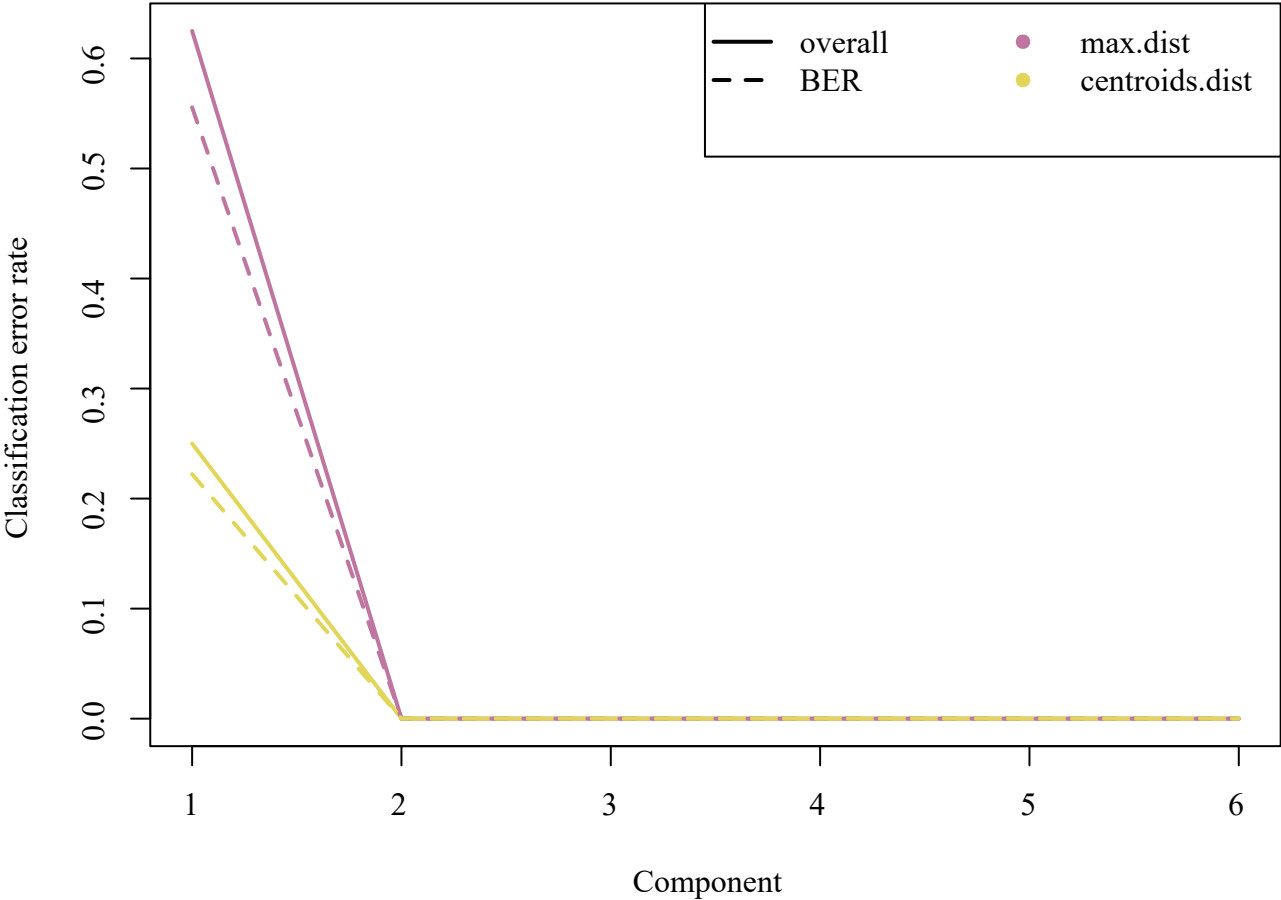

B

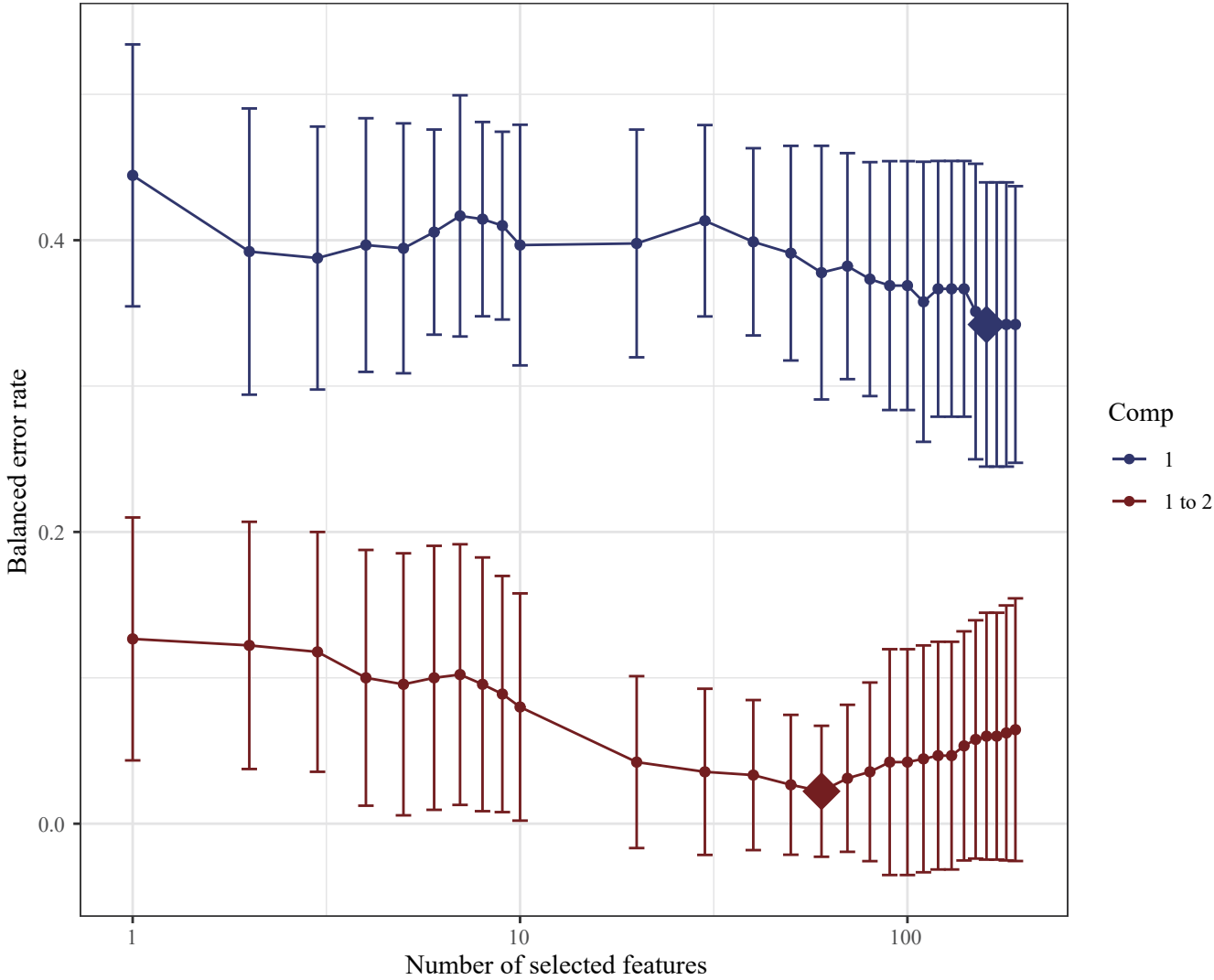

Supplement: Supplementary file 1 [file animals-14-00011-s001.zip › Figure_S2.pdf]

A

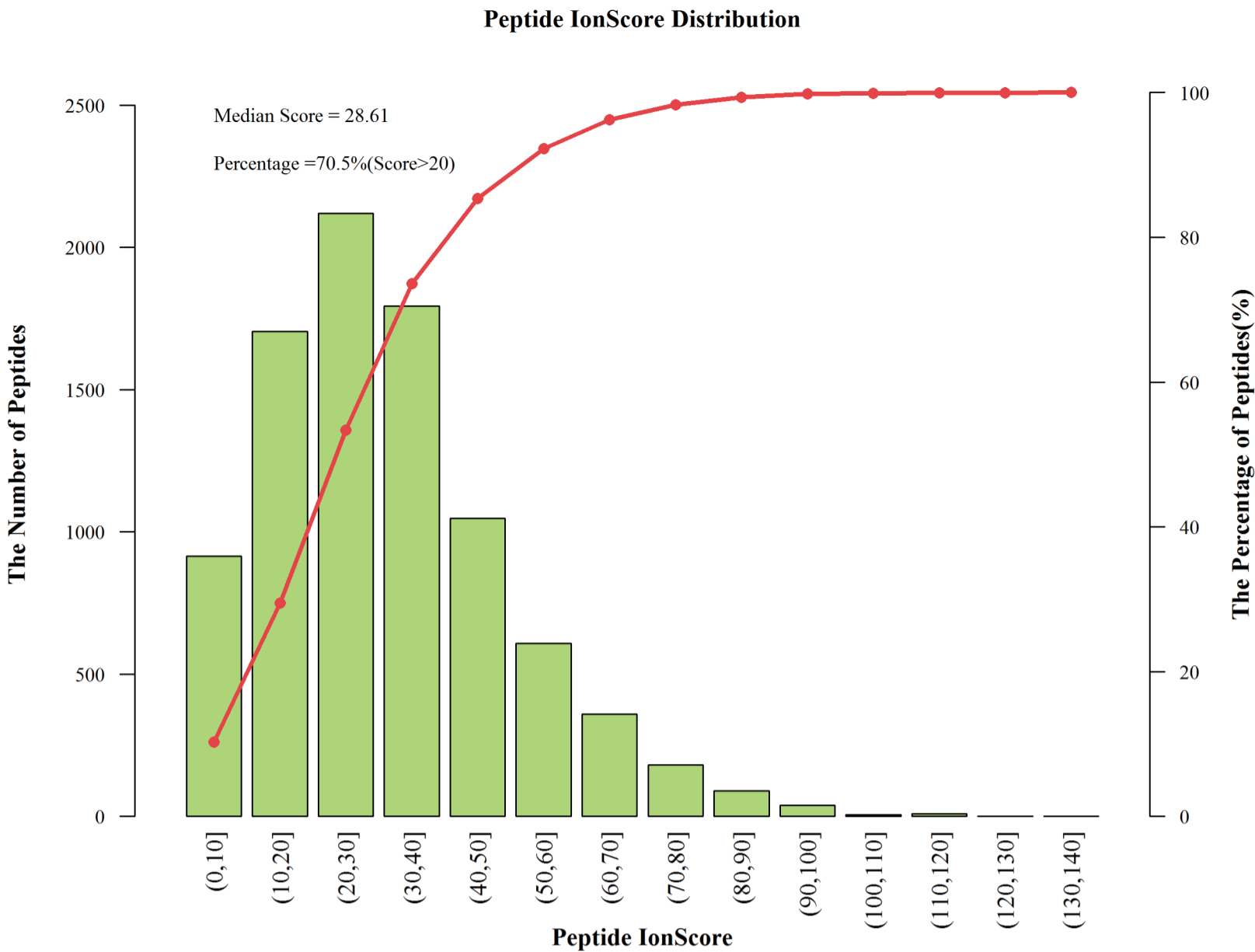

B

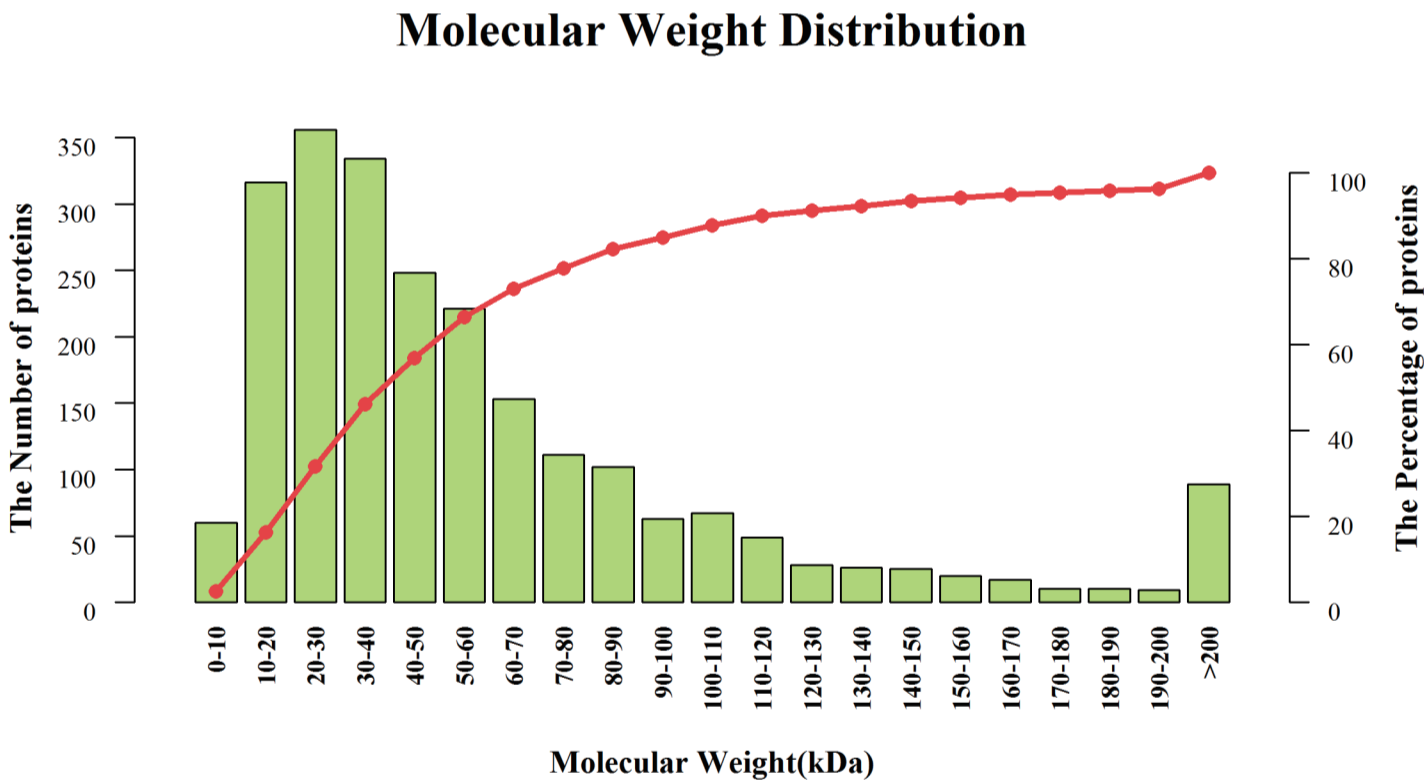

C

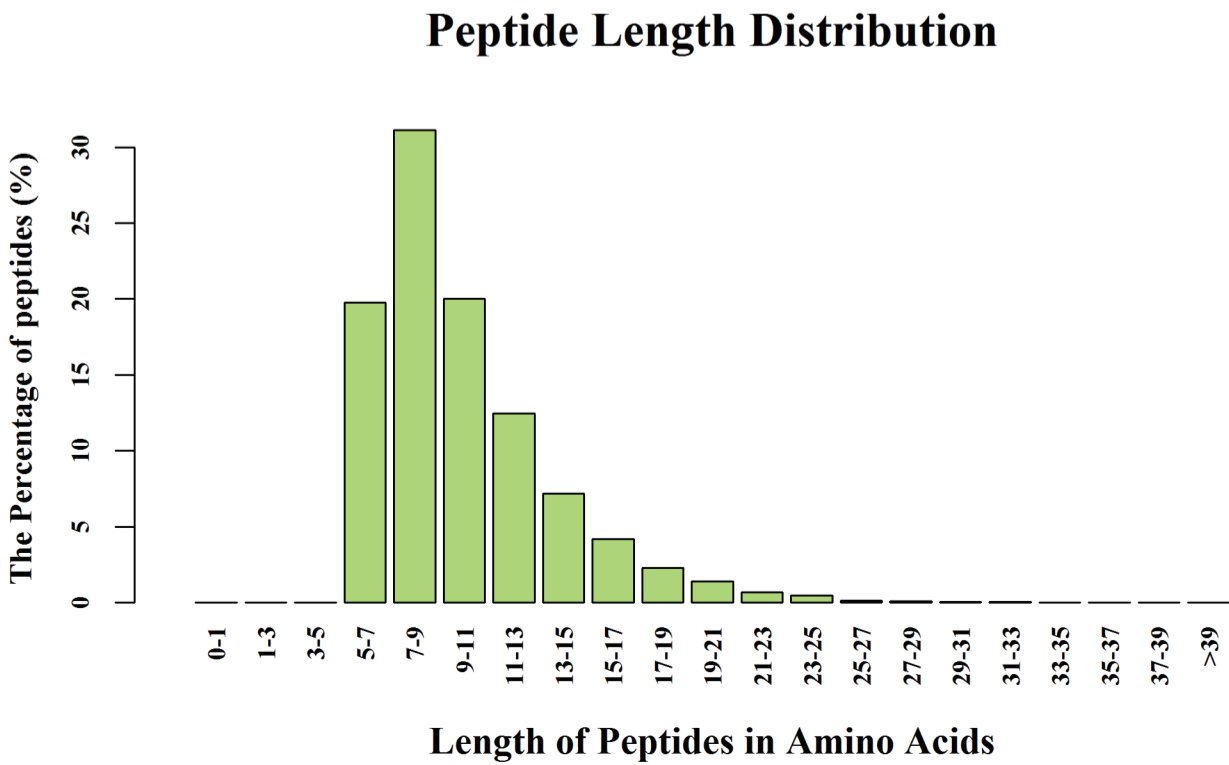

Supplement: Supplementary file 1 [file animals-14-00011-s001.zip › Figure_S1.pdf]
